# Characterization of Complex Proteoform Mixtures by Online Nanoflow Ion-Exchange Chromatography-Native Mass Spectrometry

**DOI:** 10.1021/acs.analchem.4c01760

**Published:** 2024-05-21

**Authors:** Ziran Zhai, Despoina Mavridou, Matteo Damian, Francesco G. Mutti, Peter J. Schoenmakers, Andrea F. G. Gargano

**Affiliations:** † Analytical Chemistry Group and Biocatalysis Group, Van’t Hoff Institute for Molecular Sciences (HIMS), University of Amsterdam, Science Park 904, 1098 XH Amsterdam, The Netherlands; ‡ Centre for Analytical Sciences Amsterdam, Van’t Hoff Institute for Molecular Sciences (HIMS), University of Amsterdam, Science Park 904, 1098 XH Amsterdam, The Netherlands

## Abstract

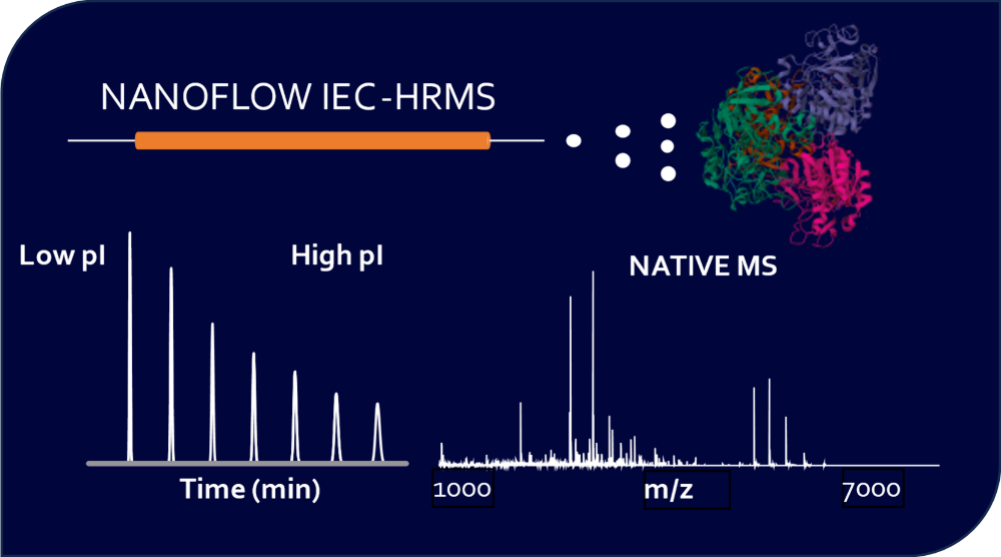

The characterization of proteins and complexes in biological systems is essential to establish their critical properties and to understand their unique functions in a plethora of bioprocesses. However, it is highly difficult to analyze low levels of intact proteins in their native states (especially those exceeding 30 kDa) with liquid chromatography (LC)-mass spectrometry (MS). Herein, we describe for the first time the use of nanoflow ion-exchange chromatography directly coupled with native MS to resolve mixtures of intact proteins. Reference proteins and protein complexes with molecular weights between 10 and 150 kDa and a model cell lysate were separated using a salt-mediated pH gradient method with volatile additives. The method allowed for low detection limits (0.22 pmol of monoclonal antibodies), while proteins presented nondenatured MS (low number of charges and limited charge state distributions), and the oligomeric state of the complexes analyzed was mostly kept. Excellent chromatographic separations including the resolution of different proteoforms of large proteins (>140 kDa) and a peak capacity of 82 in a 30 min gradient were obtained. The proposed setup and workflows show great potential for analyzing diverse proteoforms in native top-down proteomics, opening unprecedented opportunities for clinical studies and other sample-limited applications.

There is a great diversity in critical functions associated with proteoforms, a term used to describe protein products arising from homologous genes as a result of sequence variations, alternative splicing, protein complexes, and post-translational modifications.^1−4^ Therefore, the accurate and comprehensive identification of proteoforms is crucial for fundamental studies of biological processes (e.g., cancer biology).^5−8^ However, the standard approach to characterize proteins, which is bottom-up proteomics, cannot directly identify proteoforms as the presence of proteins is inferred from peptides.^4^ Top-down proteomics and intact protein MS circumvent these limitations, allowing the direct identification and analysis of intact proteoforms. This approach has shown great potential in characterizing protein mixtures, probing cellular heterogeneity, and unveiling some underlying biological functions of proteoforms.^9−12^ However, the most common top-down methods use denaturing RPLC-MS approaches, under conditions in which proteins are unfolded and noncovalent protein complexes are lost.^13^ Yet, when analyzing complex protein mixtures such as cell lysates, (i) molecular weight (MW) fractionation before analysis needs to be performed,^14^ and (ii) only the limited proteoforms identification above 30–60 kDa can be obtained.^15,16^

An alternative to denaturing MS is native MS. In native MS, water-based solutions using volatile buffers at neutral pH are employed, operating under conditions where proteins are non-denatured and labile protein complexes can be preserved.^17^ To date, most studies that apply native MS use purified samples and direct infusion with nanospray sources. This limits the applications of native MS to the analysis of nonpurified complex mixtures.^18,19^ Therefore, there is a pressing need for novel strategies to directly analyze complex mixtures of intact proteins and complexes with a wide range of molecular weights by native LC-MS approaches. Also, it is imperative to achieve high MS sensitivity, given the low abundance of target proteins in biological samples.^5,20^

Hyphenating native separations to native MS allows the measurement of complex samples, resolving proteoforms according to specific mechanisms (therefore aiding identification) and increasing the dynamic range of the measurement. Cation-exchange chromatography (CEX), in particular, is a nondenaturing separation mode that is considered the benchmark method for separating charge variants of proteins.^21,22^ Minor modifications of proteins often result in changes of iso-electric points (pI), resulting in alterations of their surface-charge distributions and, therefore, influencing the retention in CEX.^23,24^ To obtain a detailed characterization of native proteins, CEX can be coupled to high-resolution MS.^25,26^ The use of volatile salts facilitates direct coupling of CEX to MS.^27,28^ This approach has been successfully applied to the characterization of biotechnological protein products available in relatively large amounts (e.g., several tens of μg of purified biopharmaceuticals) but is not sensitive enough for broad application to biological systems.^29,30^

In this work, we have developed a method to characterize intact proteins and complexes in their native states, covering a broad MW range with a small sample consumption (down to 33 ng injection for reference monoclonal antibodies). In our approach, we directly coupled nanoflow (250 or 500 nL min^–1^) strong cation exchange chromatography (SCX) to nanoelectrospray-ionization (nESI) under native MS conditions. Proteins were separated on packed capillary SCX columns and eluted according to their pI values. High contents of volatile salts (up to 250 mM) were exploited in combination with a pH gradient (from pH 5.0 to 8.5) to ensure elution. Such a combined elution strategy is referred to as the salt-mediated pH gradient.^31,32^ To perform nESI-MS, a nano-emitter coated with a hydrophobic material was used.^33^ The use of this emitter type was crucial to avoid clogging of the flow path by the salts. The low flow promoted desolvation/ionization efficiency, allowing for sensitive detection of low-abundant proteins and complexes. We successfully applied our method to analyze an *E. coli* cell lysate and observed hundreds of proteins with masses up to 150 kDa. We believe the proposed nanoSCX-nMS is a promising approach for characterizing proteoforms and provides a universal strategy to overcome detection limitations in native top-down proteomics. In particular, our method, with respect to state-of-the-art RPLC-MS methods,^19^ (i) analyzes proteins under non-denaturing conditions, allowing the study of protein complexes and (ii) allows to analyze complicated protein mixtures distributed over wide MW ranges without the need for MW fractionation.^34^ Compared with native methods such as native capillary zone electrophoresis,^35,36^ it realizes high-performance separations for proteins with a wide range of pI values as well as concentrates the sample before the analysis, thereby increasing the sensitivity of the measurement.

Our research was inspired by the work carried out at an analytical scale in the characterization of charge variants of monoclonal antibodies (mAbs) by SCX-MS.^29^ In particular, an SCX column was used to develop an analytical flow UV-based separation method. Proteins with different properties, i.e. ribonuclease A (RNase-A), carbonic anhydrase (CA), myoglobin (Myo), bovine serum albumin (BSA), pembrolizumab (Pem), cetuximab (Cet), and trastuzumab (Tra) (for details, see Supporting Information Table S1), were employed to test different elution conditions and evaluate the performance of the methods.

To develop our method, we first performed a set of experiments to optimize a salt-mediated pH-gradient method over a broad pH range, aiming to apply this method to analyze proteins with pI values of 5 and above. To establish optimum conditions, gradients ranging from pH 5.0 to a value between 8.0 and 10.0 were employed, incorporating initial salt concentrations of either 20 mM or 50 mM and elution mobile phase concentrations from 140 mM to 250 mM. Figure S1 indicates that a higher final pH (above 9.0) reduces resolution for mixtures of proteins and mAbs, especially for analytes with high pI values (Myo and RNase-A). This presumably relates to the nonlinear pH gradient that results in an abrupt change of pH above 7, quickly changing the in-solution charge state of the proteins. In contrast, increasing the concentration of salts (pH 8.5) allowed the model proteins Myo and RNase-A to be completely separated (Figure S2). In addition, the slightly higher initial concentration of ammonium acetate (AmAc, 50 mM) yielded a better separation. By monitoring the pH profile (Figure S3), we succeeded in creating a more linear pH change zone (over 18 min) that started from 50 mM AmAc at pH 5.0 (adjusted with acetic acid) to 250 mM AmAc at pH 8.5 (adjusted with ammonia). This method was employed for all subsequent measurements.

Next, the analytical column was unpacked to collect the stationary phase and prepare capillary SCX columns (150 mm × 75 or 100 μm I.D.) and trap columns (40 mm × 150 μm I.D.) Details on the packing procedures are in Table S3. The nanoSCX-nMS setup is reported in Figure S4. The use of nanospray in the nanoSCX-nMS system allows for circumventing the desolvation stage with heated nitrogen gas typically present in capillary and analytical flow setups, thus limiting protein denaturation. The improved desolvation efficiency of the nanospray also leads to an enhancement of the sensitivity.^37,38^

To investigate whether our method was sufficiently sensitive, we analyzed small amounts of BSA (0.1 μg to 1.0 μg injected on column). The MS intensity (Figure S5) of the extracted ion chromatograms over three charge states varied from 2.4 × 10^7^ (for 0.1 μg) to 7.0 × 10^8^ (for 1 μg). Mixtures of proteins and mAbs (0.1 mg mL^–1^) were used to evaluate the separation performance of the homemade capillary SCX columns. BSA, CA, and RNase-A were well separated and eluted in the order of increasing pI (Figure 1a). Pem, Cet, and Tra (pI between 7.6 and 9.1) were also separated (Figure 1b). To further increase the sensitivity of our method, a trap column was employed to preconcentrate the low-concentration, high-volume samples. Such focusing avoids undesired peak broadening and loss of resolution in the nanoflow regime when injecting large volumes. Figure 1c shows a comparison of a trap injection of 10 μL of mAbs mixture with a concentration of 0.01 mg mL^–1^ and a trap-less injection of 1 μL of the same mixture with a concentration of 0.1 mg mL^–1^. Clearly, using a focusing trap creates possibilities to analyze amount-limited complex samples without sacrificing the separation performance.

**Figure 1:**
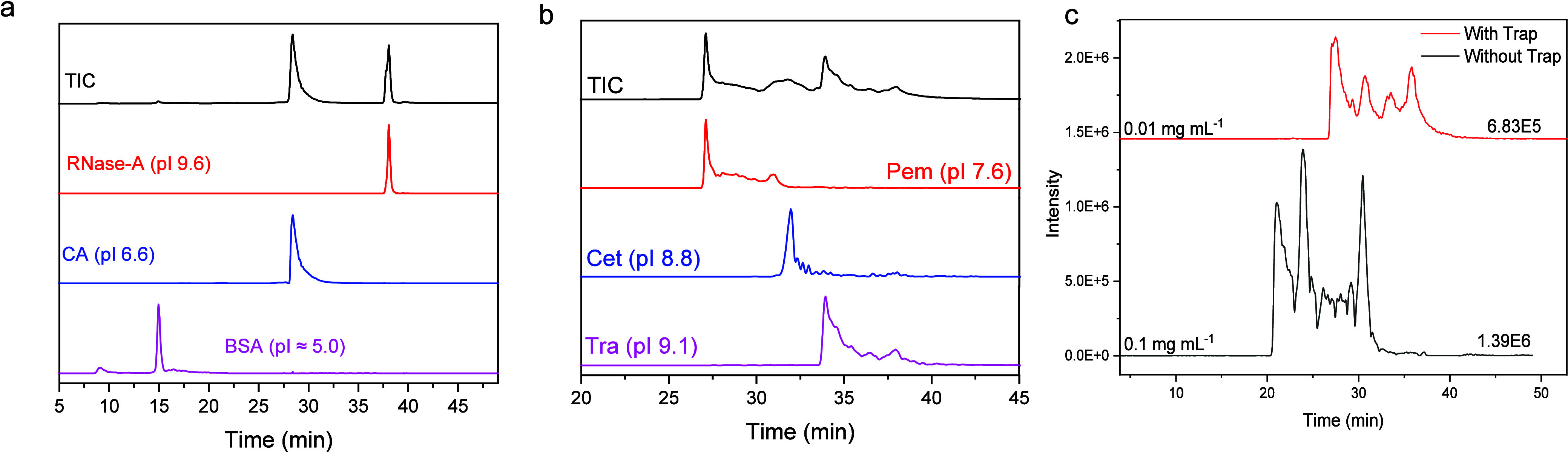
Nanoflow cation exchange chromatography-mass spectrometry analysis of (a) a mixture of model proteins: ribonuclease A, carbonic anhydrase, and bovine serum albumin. (b) Analysis of a mAbs mixture: pembrolizumab, cetuximab, and trastuzumab. The theoretical pI values are reported in the labels. The *m*/*z* values used for the extracted ion chromatograms (EIC) are reported in Table S2. (c) Total ion chromatograms in the mass range 5000–8000 *m*/*z* from the mAbs mixture with and without using a trap column to concentrate the analytes. The intensity and concentration are reported above the traces.

From the low number of charge states of proteins and mAbs in MS spectra (shown in Figures S6 and S7), we conclude that the proteins remained in their native or nondenatured states during the entire analysis process.^39^ To further evaluate the denaturation induced by our method, we analyzed model oligomeric proteins: alcohol dehydrogenase (ADH, tetramer) and l-asparaginase (monomer to octamer). The mild conditions applied retained the complexes during the analysis (Figure S8).

Next, the *E. coli* cell lysate was analyzed and used to evaluate the performance of the trap-based nanoSCX-nMS method. The sample is a complex mixture of proteins with a broad range of MW as seen by denaturing SDS-PAGE (Figure 2a). The approximate concentration measured by Bradford assay was about 9.5 mg mL^–1^ of which 5 μL were injected (about 50 μg on the column, Figure S9; note that acidic proteins may be lost when loading the sample on the trap column). In the total ion chromatogram (TIC, Figure 2b), numerous peaks were distributed across a broad time range (10 to 37 min) and high MS intensities (up to 8.0 × 10^8^) were observed. The majority of the protein feature observed had MWs above 30 kDa, as shown in Figures 2 and 3. The spread of intensities and retention times indicate a broad range of proteins present in the cell lysates, both in terms of abundance and pI values. The analysis of the average peak width of the 6 proteoforms extracted in Figure 2c (with MW from 30 until 140 kDa revealed; more EIC are shown in Figure S10) resulted in a peak capacity of about 82 with a gradient of 30 min (Table S5). These proteins retained their native structures as judged by their mass spectra (Figure 3a through 3f). Notably, our method can resolve large proteoforms (around 141 kDa) differing in mass by only 54 Da (Figure 3e and 3f, peaks 5 and 6). Moreover, the protein charge state distributions approach the maximum *m*/*z* reachable with the tested mass spectrometer (8000 *m*/*z*). Results on 15 other representative peaks with MW ranging from around 10 kDa to 100 kDa are shown in Figure S10 and Table S6. About 140 deconvoluted masses between 10 and 140 kDa could be observed applying deconvolution and are plotted in Figure 3g and Figure S11.

**Figure 2:**
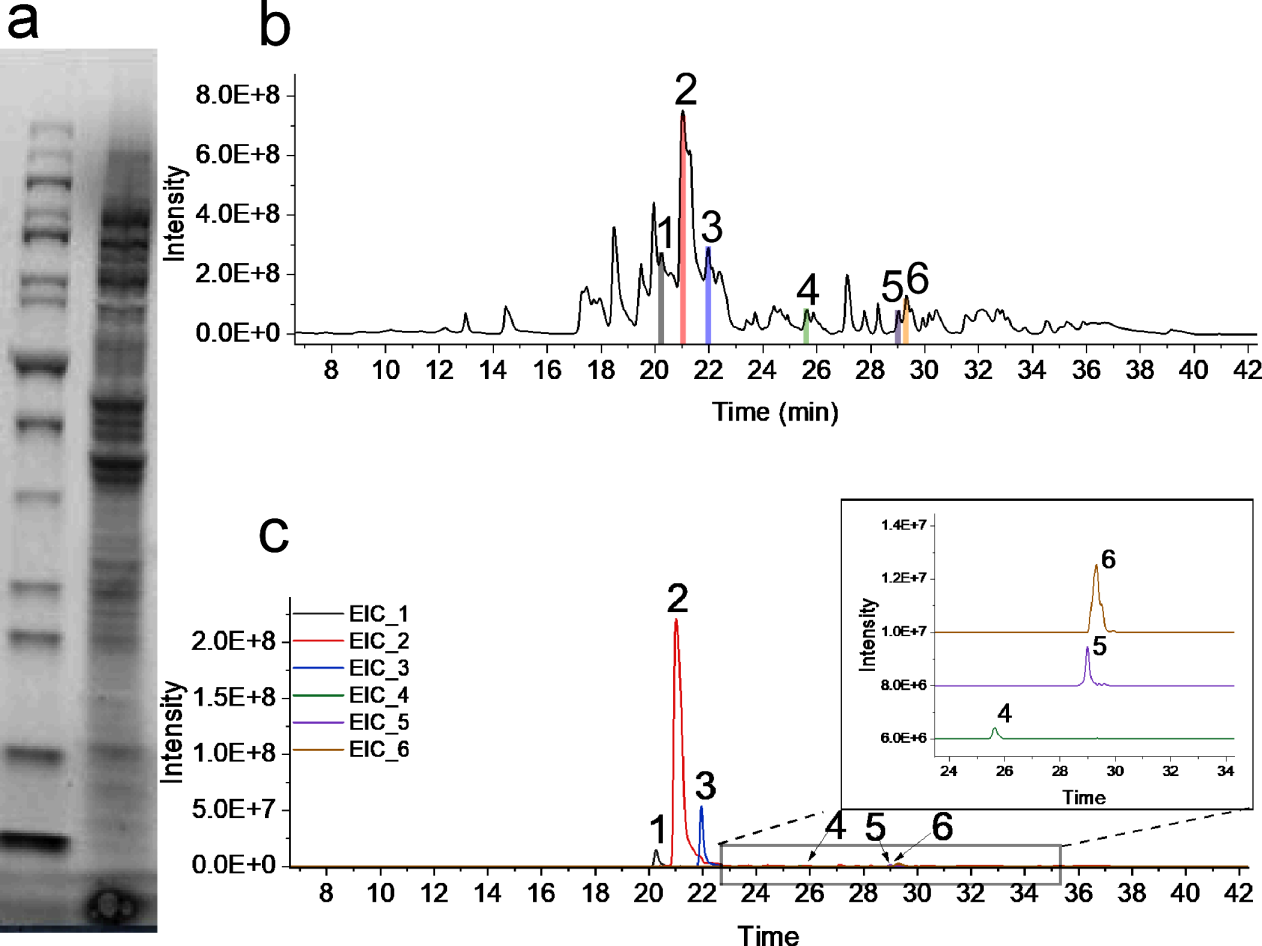
(a) SDS-PAGE analysis of the *E. coli* cell lysates (bands on the right) with respect to a protein MW ladder (left) illustrates the molecular distribution and complexity of the cell lysate sample. (b) TIC of *E. coli* cell lysate analyzed by the nanoSCX-nMS. (c) EIC of labeled peaks in (b). The *m*/*z* values used to extract these peaks and corresponding MS spectra for the EIC are reported in Figure 3 and Table S4.

**Figure 3:**
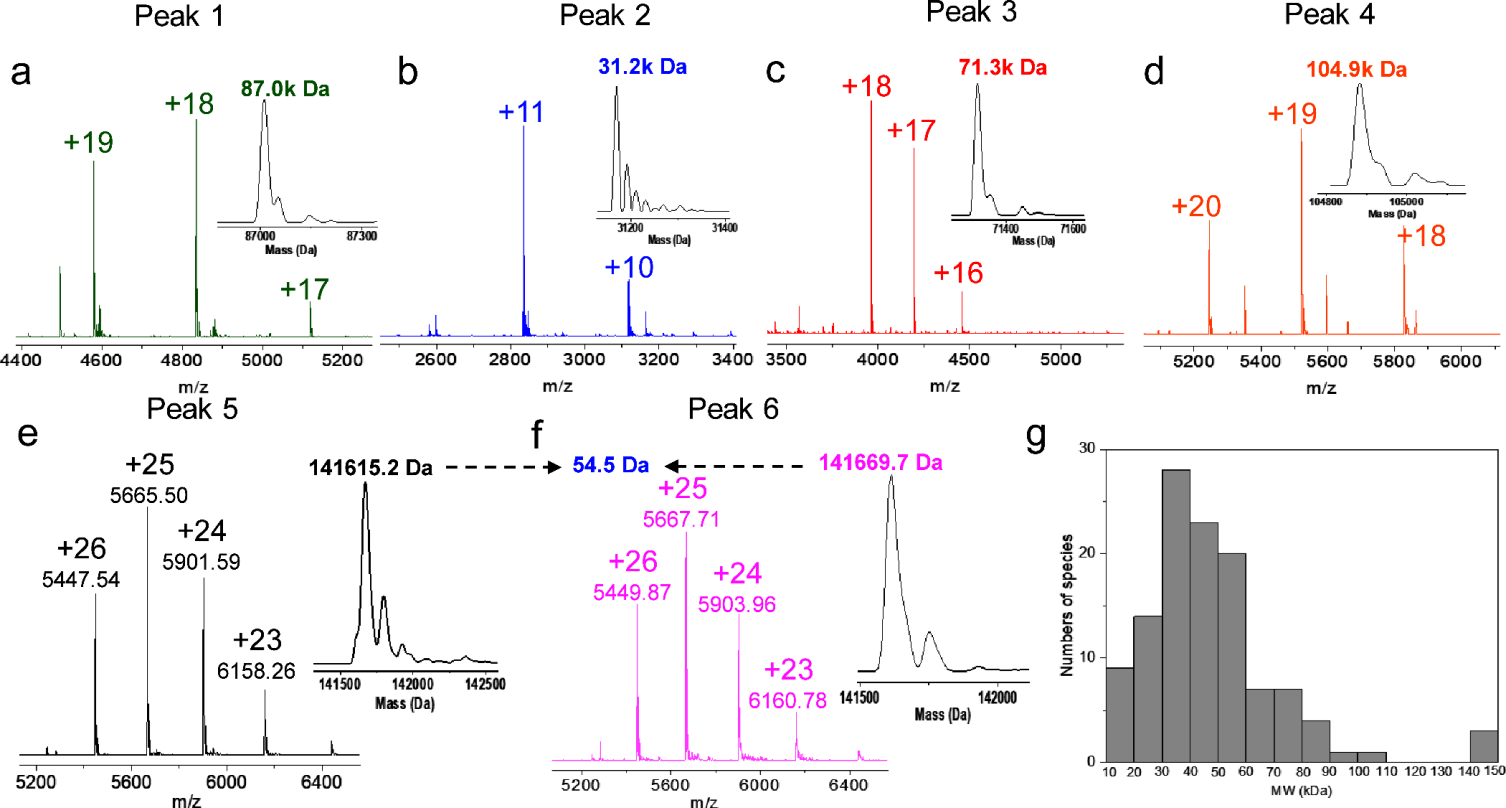
(a–f) Mass spectrum and deconvoluted spectra of peak 1 to peak 6 reported in Figure 2. (g) Histogram describing the distribution of the deconvoluted masses identified in the nanoSCX-nMS analysis of the *E. coli* cell lysate reported in Figure 2. Details on deconvolution parameters are provided in the Supporting Information.

In conclusion, we developed a novel approach for online nanoflow strong cation exchange chromatography with native MS detection. The method allowed rapid and accurate analysis of proteoforms under native conditions with good repeatability (e.g., <2% shift in retention time between repetitions when analyzing protein standards). The high sensitivity of the method, resulting from the high desolvation efficiency in nESI and the trap-and-elute preconcentration setup, suggests a great potential for the characterization of low-abundant species. Maintaining the native states of proteins leads to simpler mass spectra with reduced numbers of charge states with respect to denaturing MS, which is beneficial for the characterization of complex protein mixtures with broad MW ranges without prefractionation. The nanoSCX-nMS method provides a streamlined and efficacious approach for analyzing proteoforms by native top-down proteomics, with far-reaching applications to help elucidate the roles of proteins in biological processes.

## Supporting Information Available

Details of materials and methods; optimization of salt-mediated pH gradient; pH profile of obtained gradient method; preparation of capillary columns; workflow of nanoSCX-nMS setup; sensitivity test with various concentrations of BSA; measurements of proteins, mAbs, protein complexes, and *E. coli* cell lysates by nanoSCX-nMS; MS spectra of proteins and mAbs mixtures; Bradford method to test the concentration of cell lysate; deconvolution results of *E. coli* cell lysates; tables of summarizing information on proteins, mAbs, column-packing conditions, *m*/*z* values for EIC, and FWHM for extracted peaks (PDF)
