# Supplementary material for: Characterization of Complex Proteoform Mixtures by Online Nanoflow Ion-Exchange Chromatography-Native Mass Spectrometry
Source: Anal Chem. 2024 May 21;96(22):8880–5. doi: 10.1021/acs.analchem.4c01760 (PMC11154664; doi:10.1021/acs.analchem.4c01760)
Supplement: Supplementary file 1 — ac4c01760_si_001.pdf [file ac4c01760_si_001.pdf]

Supplementary Information

Characterization of Complex Proteoform Mixtures by Online Nanoflow Ion-Exchange Chromatography - Native Mass Spectrometry

Ziran Zhai,[†‡*] Despoina Mavridou[†‡], Matteo Damian,[†] Francesco G. Mutti,[†] Peter J. Schoenmakers,[†‡] Andrea F.G. Gargano[†‡*]

[†]Analytical Chemistry Group and Biocatalysis Group, Van't Hoff Institute for Molecular Sciences (HIMS), University of Amsterdam, Science Park 904, 1098 XH Amsterdam, The Netherlands.
[‡]Centre for Analytical Sciences Amsterdam, Van't Hoff Institute for Molecular Sciences (HIMS), University of Amsterdam, Science Park 904, 1098 XH Amsterdam, The Netherlands.

*Corresponding author: Andrea F.G. Gargano; Ziran Zhai
E-mail: a.gargano@uva.nl; z.zhai@uva.nl

Table of Contents

1. General information........S3
   1.1 Chemicals and Materials........S3
   1.2 HPLC-UV/FLD........S3
   1.3 Nanoflow SCX - native mass spectrometry (nMS)........S3
   1.4 Data analysis........S4
2. Development of salt-mediated pH gradient method........S4
3. Preparation of capillary SCX and trap columns........S4
4. Proteins and mAbs mixtures measured by nanoflow SCX-nMS........S5
5. Measurements of *E. coli* cell lysates by nanoSCX-nMS........S5
6. Supplemental Figures........S7
7. Supplemental Tables........S12
8. Reference........S14

1. General information

1.1 Chemicals and Materials

Ammonium acetate (AmAc, ≥ 98%), ammonia solution (25%), sodium phosphate dibasic (≥ 98.5%), and sodium phosphate monobasic (≥ 99.0%) were purchased from Sigma-Aldrich (Steinheim, Germany). Acetic acid (100%) was obtained from Merck (Darmstadt, Germany). Sodium sulfate (≥ 99%) was obtained from Merck (Hohenbrunn, Germany). Water (LC-MS grade) was purchased from Biosolve (Valkenswaard, The Netherlands). Bovine serum albumin (BSA, ≥ 96%), carbonic anhydrase from bovine erythrocytes (CA, ≥ 95%), myoglobin from equine skeletal muscle (Myo, 95% - 100%), ribonuclease A from bovine pancreas (RNase-A, ≥ 60%), and alcohol dehydrogenase (ADH) from saccharomyces cerevisiae (>300 units/mg) were purchased from Sigma-Aldrich (St. Louis, USA). Trastuzumab (Tra) was obtained from Roche (Grenzach-Wyhlen, Germany). Cetuximab (Cet) was purchased from Merck (Amsterdam, The Netherlands). Pembrolizumab (Pem) was obtained from MSD (London, United Kingdom). The therapeutic enzyme L-asparaginase (ASNase, Paronal) produced by *E. coli* was provided by the Department of Pharmaceutical Analysis, Faculty of Pharmaceutical Sciences, Ghent University (Belgium). The ultrapure water (18.2 MΩcm) was produced by a Milli-Q purification system (Millipore, Bedford, MA, USA). Fused-silica capillaries (0.075-, 0.1- and 0.15-mm I.D., 0.36 mm O.D.) were purchased from CM Scientific (Silsden, UK). The frit kit (including formamide, Kasil-1, and Kasil-1624) was purchased from Next Advance (Troy, NY, USA). All other reagents used were of analytical grade and purchased from Sigma-Aldrich (Steinheim, Germany).

1.2 HPLC-UV/FLD

Analytical flow-based ion-exchange chromatography (IEC) was performed on an Agilent 1100 series Infinity HPLC system (Agilent, Waldbronn, Germany). The system included one isocratic pump (G1310A), a 1260 high-performance degasser (G4225A), an autosampler (G1329A), a column compartment (G1316A), a multi-wavelength detector (MWD, G1365B), and a fluorescence detector (FLD, G1321A). The Agilent OpenLAB CDS Chemstation Edition (version 3.2, build 3.2.0.620) software was employed to control the HPLC system. A strong cation-exchange (SCX) column BioPro IEX SF (100 mm x 4.6 mm, 5 μm particle size, YMC, Japan) with non-porous functional particles was used to separate proteins.

1.3 Nanoflow SCX - native mass spectrometry (nMS)

Nanoflow strong cation exchange chromatography (SCX) was performed on an UltiMate RSLCnano system (Thermo Fisher Scientific, Breda, The Netherlands) equipped with a high-pressure pump with microflow selector and a loading pump (NCS-3500RS), a binary nano/capillary pump (NCP-3200RS), a thermostatted column compartment (equipped with two 10-port, two-position valves) and an autosampler. Injection loops of 1 μL (without trap) and 20 μL (with trap) were used. The autosampler was kept at 7 °C during the analysis. A QExactive-Plus Biopharma high-resolution

mass spectrometer (Thermo Fisher Scientific, Bremen, Germany) was employed. Nanospray ionization was realized with a nanospray-flex-series ion-source platform (Thermo Fisher Scientific), a Simple Link UNO (1/32, Fossiliontech, Albacete, Spain), and a nano emitter (75 mm length × 20 µm I.D., Fossiliontech) with hydrophobic coating were used.

1.4 Data analysis

The data from HPLC-UV/FLD were analyzed with the Agilent OpenLAB CDS Chemstation software (offline). The data from nanoflow SCX-nMS were analyzed with Thermo Xcalibur Qual Browser (version 4.4) and Freestyle software (version 1.7, Thermo Fisher Scientific). The UniDec program (University of Arizona, Phoenix, AZ, USA) and BioPharma Finder (version 4.0, Thermo Fisher Scientific) was used to perform deconvolution of mass spectra and obtain the molecular weight (MW).[1] Total ion chromatogram (TIC), extracted ion chromatogram (EIC), and base peak chromatogram (BPC) were smoothed using a 7-point Gaussian filter. The data presented are available at the following MassIVE Repository link: ftp://massive.ucsd.edu/v07/MSV000094068/. Deconvoluted parameters for Figure 3g and S9 are as follows: Time start: 10 to 40 min; Sliding windows (min): 1; Offset: 10; Partition in time step of: 1 min; The m/z range: 1000 to 8000; Charge range: 5 to 30; Mass range: 10,000 to 150,000 Da; Sample mass every (Da): 1; Picking range (Da): 5; Picking threshold: 0.01.

2. Development of salt-mediated pH gradient method

To realize the online coupling of SCX and MS, volatile salts were used. Standard mixtures of proteins (CA, BSA, Myo, and RNase-A) and monoclonal antibodies (mAbs; Pem, Cet, and Tra) were used to evaluate the developed method. 20 mM or 50 mM AmAc (pH=5.0, adjusted by acetic acid) was used as mobile phase A (MPA). A series of concentrations of AmAc (140 mM to 250 mM) and pH (8.0 to 10.0, adjusted by ammonia) were investigated as mobile phase B (MPB). Proteins and mAbs mixtures were reconstituted with mobile phase A to a concentration of 1 mg/mL. The injection volume was 5 µL. The wavelengths of UV detection were set at 220, 256, and 280 nm. The excitation and emission wavelengths for FLD detection were 280 nm and 340 nm, respectively. The gradient started from 100% MPA and went to 100% MPB in 22.5 min, at a flow rate of 0.4 mL/min. To monitor the pH profile in the salt-mediated pH-gradient method, blank fractions were collected every 1 min, and the corresponding pH values were obtained using a pH meter.

3. Preparation of capillary SCX and trap columns

The strong cation-exchange (SCX) column, BioPro IEX SF (100 mm x 4.6 mm, 5 µm particle size, YMC, Japan), was unpacked to collect the functional resins. An empty capillary was end-sealed with a frit before use. The obtained resins were reconstituted in the packing solution, composed of 500 mM Na_2SO_4 and 50 mM PBS (pH 7.0), before they were flushed into the end-sealed capillary with a pump at a relatively stable pressure. After observing the capillary to be fully packed, the packing flow was

maintained for at least 10 min to stabilize and compact the resin. Finally, the columns were flushed with a low concentration of volatile salts before application.

4. Proteins and mAbs mixtures measured by nanoflow SCX-nMS

Mobile phases composed of 50 mM AmAc (pH=5.0 adjusted by acetic acid, MPA) and 250 mM AmAc (pH=8.5 adjusted by ammonia, MPB) were filtered through 0.4-μm micropore membranes before use. Laboratory-packed capillary SCX columns and traps were employed to do the measurements. Figure S4 shows the workflow of the nanoflow SCX-nMS setup (nanoSCX-nMS). To preliminarily investigate the sensitivity of nanoSCX-nMS system, a series of concentrations of BSA (from 0.1 mg/mL to 1.0 mg/mL) were tested. To evaluate the chromatographic performance, mixtures in MPA of proteins (RNase-A, Myo, CA, and BSA) and mAbs (Pem, Cet, and Tra) of 0.1 mg/mL were measured without a trap, while mixtures of 0.01 mg/mL were measured with a trap. A mobile phase of 20 mM AmAc (pH=4.5, adjusted by acetic acid) was used to load samples on the trap using the loading pump. The flow rate was set at 0.5 μL/min (for a 100-μm I.D. column) or 0.25 μL/min (for a 75-μm I.D. column). The injection volume was 1 μL or 10 μL, without or with a trap, respectively. The temperature of the autosampler was kept at 7 °C and no temperature control was installed for the column oven. The gradient ran from 0% MPB to 100% MPB in 30 min. Prior to measurements, the system was operated for at least 5 hours to be stable.

The MS instrument was operated at a spray voltage between 1.8 and 2.0 kV (depending on the conditions of the emitter), with a transfer-capillary temperature of 275 °C. The radio frequency of the S-lens was 200. The acquisition parameters were as follows. Scan mode, HRM; scan range, 1000 to 8000 m/z; in-source collision-induced dissociation (isCID), 55 eV for protein mixtures, and 85 eV for mAbs mixtures; number of microscans, 10; resolution, 17,500; automatic-gain-control (AGC) target, 3 x 10^6; maximum injection time (IT), 200 ms.

5. Measurements of *E. coli* cell lysates by nanoSCX-nMS

To obtain the *E. coli* cell lysate, 5.13 g of *E. coli* cells containing overexpressed Te-ADH were suspended in 50 mL of KPi buffer with a concentration of 50 mM and pH 8 (10 mL buffer for 1 g of cells). The suspension was sonicated for 10 minutes (10 s pulse ON, 10 s pulse OFF, 45% amp) and kept at 0 °C. Next, the resulting suspension was centrifuged at 4 °C and 14,000 rpm for 60 min. Finally, the supernatant was filtered (0.45 μm filter) and collected for analysis.

Firstly, 40 mL solutions of *E. coli* cell lysate were freeze-dried. Then the residue was reconstituted with 4 mL of 20 mM AmAc. After centrifuging the obtained solution at 5 °C and 12,000 rpm for an hour, the supernatant was collected, after which it was centrifuged again at the same conditions. Next, the obtained clear supernatant was concentrated with spin filters (cut-off MW: 3 kDa) at 5 °C and 12,000 rpm for 2 to 3 hours. The acquired solution was stored at -20 °C.

Before measurements, the pre-processed *E. coli* cell lysates were diluted tenfold with MPA. Then the formed precipitants (possibly induced by changes in pH in the samples) were removed by centrifuging at 5 °C and 12,000 rpm for 30 min. Next, the supernatant was collected and analyzed directly by trap-based nanoSCX-nMS. The injection volume was 10 µL. The other parameters were the same as for the measurements of protein and mAbs mixtures.

The Bradford method was used to test the approximate concentration of the total amount of proteins in *E. coli* cell lysate. A series of concentrations of BSA (0, 0.05, 0.1, 0.2, 0.3, 0.4, 0.5, 0.6, 0.8, 1.0 mg/mL) was prepared with KPi buffer (50 mM, pH=8.0). Then 20 µL of each concentration of BSA were mixed with 1 mL of the dye reagent correspondingly before incubating the mixtures for 5 min at room temperature. Next, the standard curve was plotted after the optical densities (OD) of the mixtures were measured at the wavelength of 595 nm. The *E. coli* cell lysate was diluted tenfold, and the concentration of it was obtained with the OD measurements (measured 0.61) and standard curve.

6. Supplemental Figures

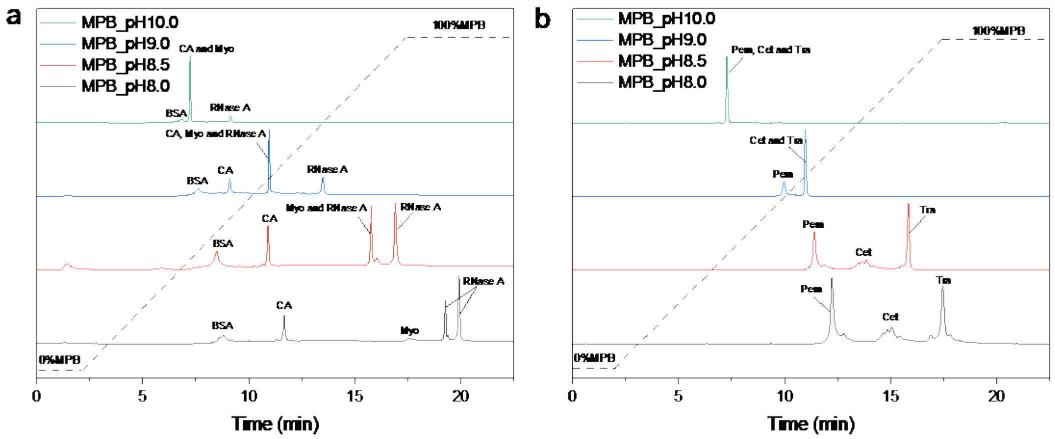

Figure S1. pH optimization for salt-mediated pH-gradient method. (a) Chromatograms of protein mixtures (normalized). (b) Chromatograms of mixtures of mAbs (normalized). MPB = mobile phase B.

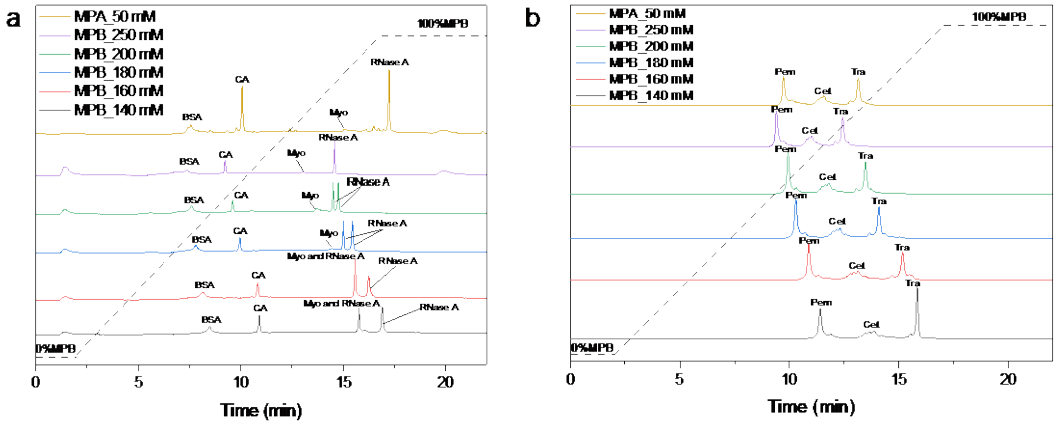

Figure S2. Optimization of the concentration of salts (pH 8.5) for salt-mediated pH-gradient separations. (a) Chromatograms of protein mixtures. (b) Chromatograms of mixtures of mAbs. MPA = mobile phase A.

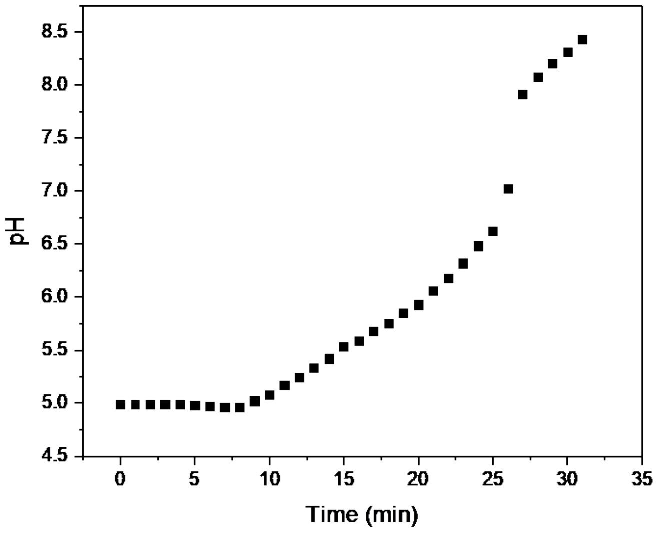

Figure S3. pH profile of the salt-mediated pH-gradient method. MPA: 50 mM AmAc (pH 5.0). MPB: 250 mM AmAc (pH 8.5). Gradient method: 0-5 min, 100% A; 5-25 min; 100% A to 100% B.

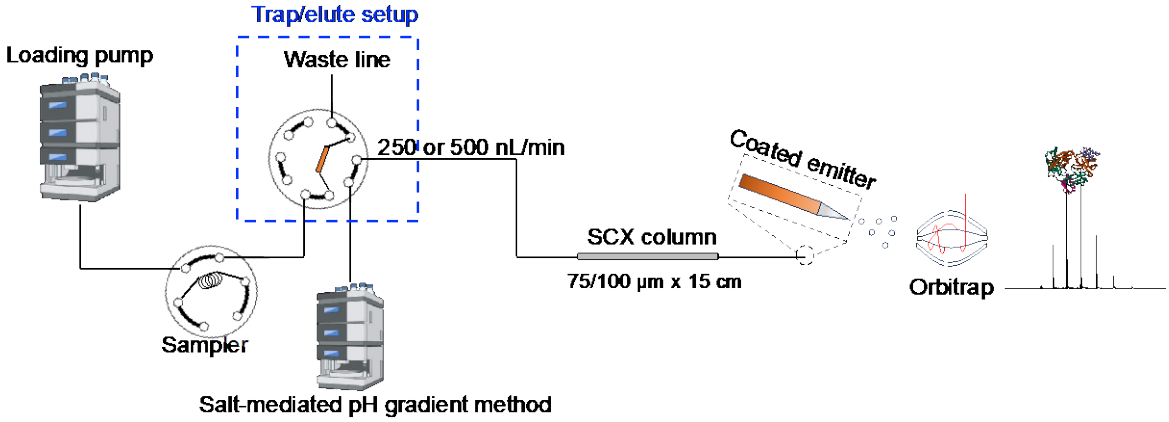

Figure S4. Workflow of nanoflow strong cation-exchange chromatography - nMS.

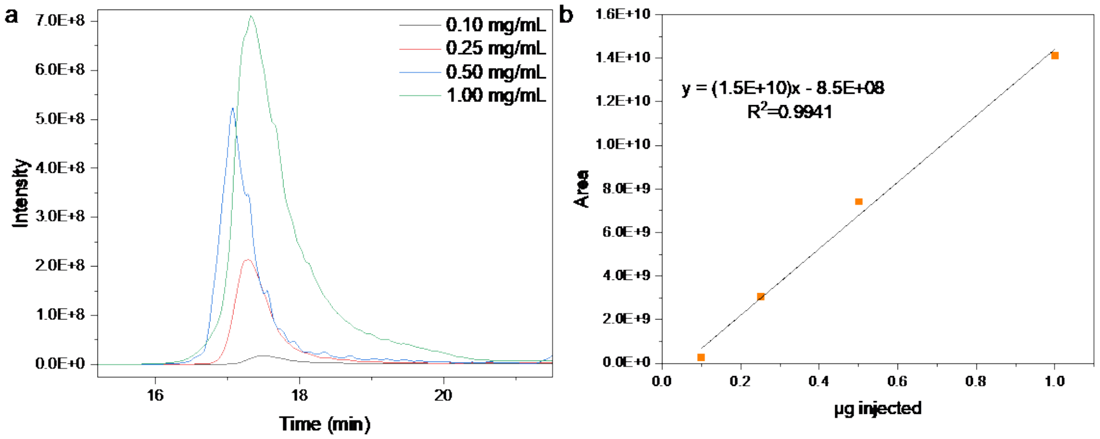

Figure S5. (a) EIC of BSA with different concentrations (0.1 mg/mL to 1 mg/mL with 1 μL injection). The m/z values used in EIC are 3908.42,4152.76,4429.53,4745.81. (b) The plot of relationship between the μg injection of BSA and corresponding peak areas.

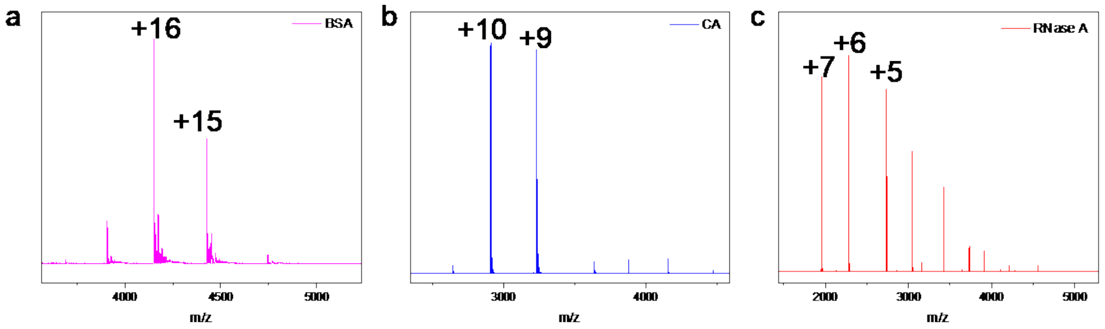

Figure S6. Mass spectra of proteins obtained with nanoflow SCX-nMS. (a) BSA; (b) CA; (c) RNase A.

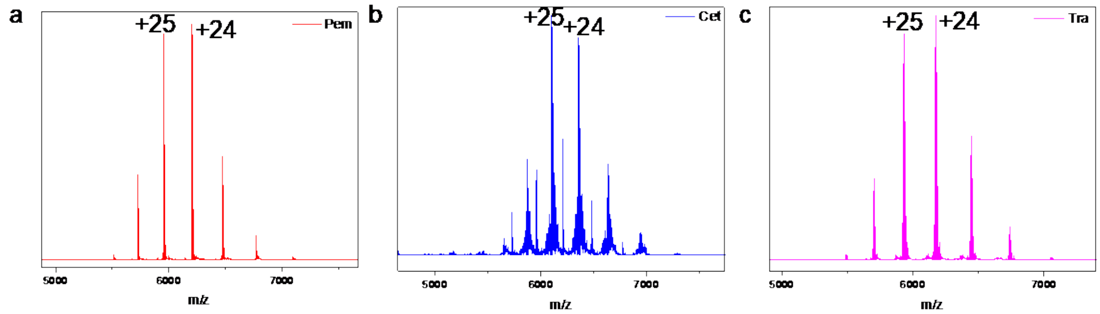

Figure S7. Mass spectra of mAbs obtained with nanoflow SCX-nMS. (a) Pem; (b) Cet; (c) Tra.

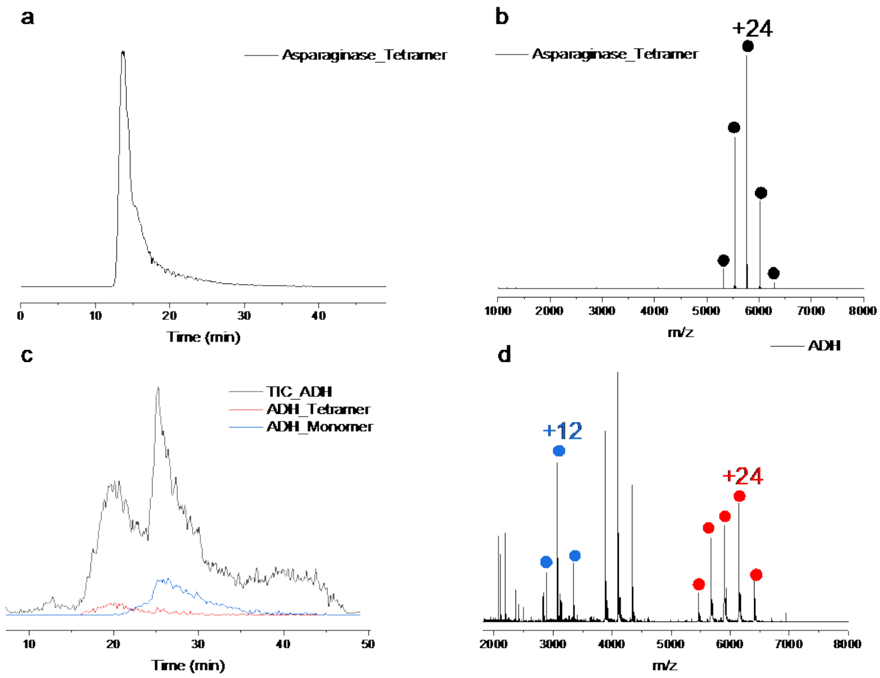

Figure S8. The analysis of protein complexes (asparaginase and ADH) with nanoSCX-nMS method. EIC (a) and mass spectrum (b) of the asparaginase. Separation (c) and mass spectrum (d) of the ADH. Concentrations of asparaginase and ADH: 0.1 mg/mL. Injection: 1 μL.

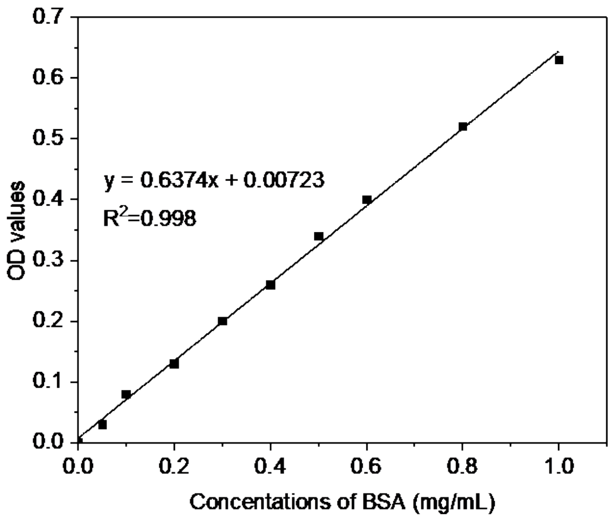

Figure S9. The standard curve of BSA measured with the Bradford method. The concentration of the *E. coli* cell lysate was obtained by the equation of $\frac{0.61-0.00723}{0.6374} \times 10$, namely 9.457 mg/mL.

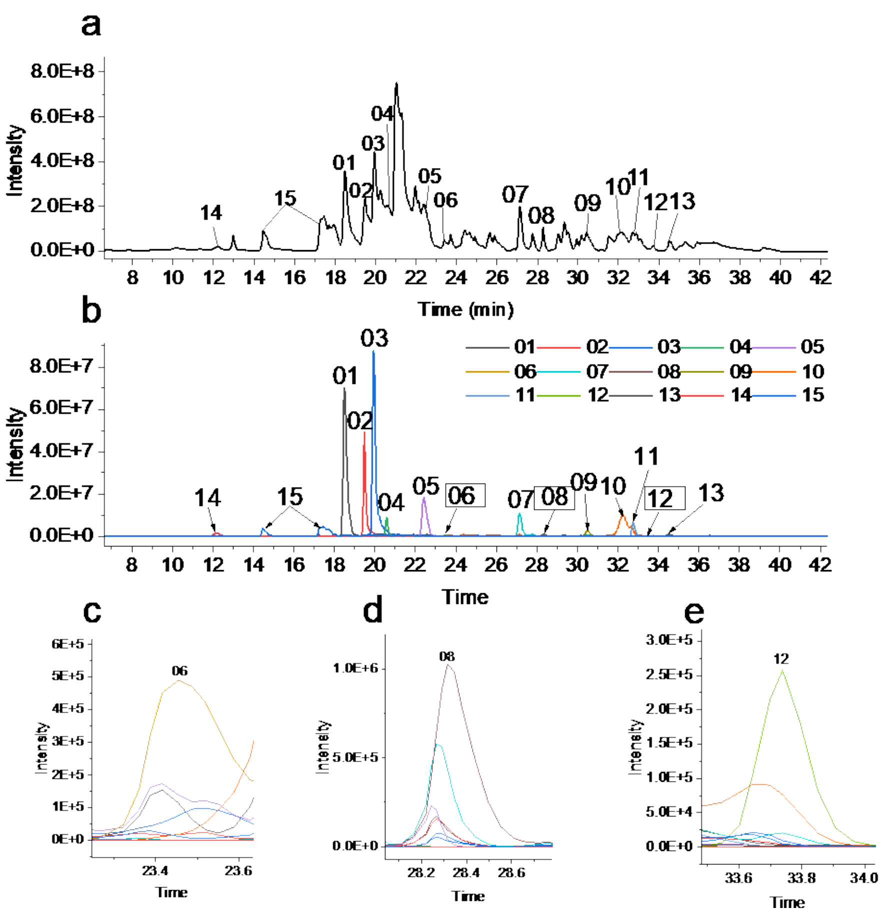

Figure S10. Nanoflow SCX-nMS run of *E. coli* cell lysate. (a) TIC of *E. coli* cell lysate. (b) EIC of the labelled peaks in (a). Figures (c), (d), and (e) show enlargements of peaks 06, 08, and 12, respectively.

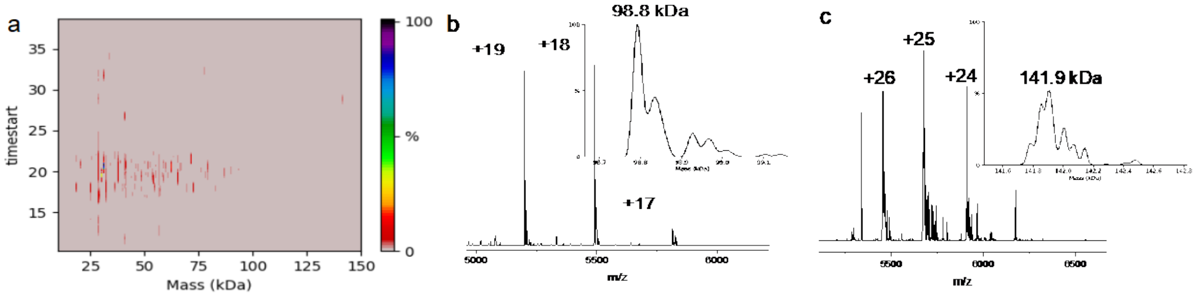

Figure S11. (a) 2D plot of deconvolution results of the *E. coli* cell lysate with UniDec. Deconvolution parameters are reported in the data analysis section. (b and c) Average mass spectra and deconvoluted spectra of the high-MW species.

7. Supplemental Tables

Table S1. Basic information on proteins and mAbs.

Proteins/mAbs	pI	MW (kDa)
BSA	4.7 – 5.6	66.4
CA	6.6	29
Myo	Around 7	16.9
RNase-A	9.6	13
Pem	7.6	146
Cet	8.8	152
Tra	9.1	148

Table S2. Lists of m/z values for EIC in Figure 1.

Peak	m/z values for EIC
BSA	3908.47, 4152.76, 4465.46, 4775.61
CA	2909.76, 3232.98, 3636.88, 2645.22
RNase-A	1955.44, 2281.47, 2737.31, 3041.43
Pem	5728.01, 5957.31, 6205.65, 6475.31
Cet	5873.31, 6107.72, 6363.01, 6632.74
Tra	5701.77, 5929.90, 6176.86, 6445.43

Table S3. Parameters for packing SCX columns and trap columns.

Columns	I.D.	Length	Packing solvents	Resin	Concentration of resin	Packing pressure
SCX	75/100 μm	15 cm	50 mM PBS and 500 mM Na_2SO_4 (pH=7.0)	Strong-cation-exchange resins (non-porous, 5 μm)	30 mg/mL	Around 120 bars (Max.150 bars)
Trap	150 μm	4 cm	50 mM PBS and 500 mM Na_2SO_4 (pH=7.0)	Strong-cation-exchange resins (non-porous, 5 μm)	30 mg/mL	Around 120 bars (Max.150 bars)

Table S4. Lists of m/z values for EIC in Figure 2.

Peak	m/z values for EIC
1	4580.58, 4834.99,5119.33
2	2834.72, 3118.00
3	3962.94, 4196.10, 4458.31
4	5245.08, 5521.15, 5827.87
5	5449.87, 5667.71, 5903.96, 6160.78
6	5447.54, 5665.50, 5901.59, 6158.26

Table S5. Lists of the full width at half maximum (FWHM) of the peaks in Figure 2c.

Peak	FWHM (min)
1	0.166
2	0.318
3	0.149
4	0.200
5	0.151
6	0.321

Table S6. Lists of m/z values for EIC and the corresponding MW of the species in Figure S8.

Peak	m/z values for EIC	Deconvolution MW (Da)
1	2018.76, 2271.25, 2595.28	18161
2	3597.26, 3854.13, 4150.48	53944
3	2754.73, 3030.13, 3366.68	30292
4	4720.96, 4983.13, 5276.22	89679
5	2009.37, 2260.52, 2583.17	18076
6	5200.58, 5489.43	98793
7	3153.40, 3416.60, 3727.13	40987
8	4710.88, 5005.26	80069
9	3959.98, 4223.91, 4525.73, 4873.79	63345
10	2817.17, 3098.82, 3443.04	30979
11	4308.26, 4561.61, 4846.68	77531
12	4155.75, 4415.45	70631
13	4123.38, 4380.95	70080
14	3772.72, 4087.01	49032
15	2497.28, 2777.04	24963

Note: the mass tolerance is within 50 ppm.

8. Reference

(1) Marty, M. T.; Baldwin, A. J.; Marklund, E. G.; Hochberg, G. K.; Benesch, J. L.; Robinson, C. V. Bayesian deconvolution of mass and ion mobility spectra: from binary interactions to polydisperse ensembles. *Anal. Chem.* **2015**, *87* (8), 4370-4376.

Authors contributions

Ziran Zhai: Conceptualization, Methodology, Investigation, Writing - Original Draft, Writing - Review & Editing. Despoina Mavridou: Methodology, Investigation. Matteo Damian: Methodology, Investigation, Resources. Francesco Mutti: Methodology, Resources. Peter J. Schoenmakers: Writing - Original Draft, Writing - Review & Editing. Andrea F.G. Gargano: Conceptualization, Methodology, Investigation, Project administration, Supervision, Resources, Writing - Original Draft, Writing - Review & Editing
